# Linking distributed leadership to teachers’ innovation: Chain mediating roles of commitment and collaboration in Chinese schools

**DOI:** 10.1371/journal.pone.0333118

**Published:** 2025-09-24

**Authors:** Yu Zhao, Xiaoyang Li, Hongqin Kang

**Affiliations:** 1 School of Education, Qufu Normal University, Qufu, Shandong, China; 2 School of Humanities and Education, Guangzhou Institute of Science and Technology, Guangzhou, Guangdong, China; 3 Faculty of Education, Qufu Normal University, Qufu, Shandong, China; University of Johannesburg, SOUTH AFRICA

## Abstract

Teachers’ innovative pedagogical practices are central to enhancing professional development and improving instructional effectiveness. However, empirical studies investigating the association between distributed leadership and teachers’ innovative practices were limited in the Chinese educational context. This study examines the associations between distributed leadership and teachers’ innovative practices, with teacher commitment and teacher collaboration considered as potential mediators. Through structural equation modeling analysis of the Teaching and Learning International Survey (TALIS, n = 3,772) 2018 data, the findings indicate that distributed leadership is significantly linked to teachers’ innovative practices. Furthermore, teacher commitment and teacher collaboration exert a significant mediating effect between distributed leadership and teachers’ innovative practices, demonstrating a chained mediation effect. The findings underscore the necessity of highlighting the need for decentralized leadership, enhanced teacher commitment, and structured collaboration to support educational innovation. This study contributes to the theoretical understanding of distributed leadership and provides actionable strategies for enhancing teacher professionalism and supporting educational reform initiatives.

## 1. Introduction

High-quality teaching is critical to improving student academic achievement. As key actors in education, teachers enhance student outcomes by continuously refining and innovating their pedagogical approaches [1]. However, teachers’ innovative practices are shaped by multiple factors, such as principal-distributed leadership, teacher commitment, and teacher collaboration [2–4].

The imperative to enhance teachers’ innovative practices has prompted growing scholarly interest in the relationship between principal-enacted distributed leadership and teachers’ pedagogical innovation [5–7]. In China, the Opinions on Comprehensively Deepening the Reform of Teacher Team Construction in the New Era outlines a vision for 2035, aiming to cultivate a stronger sense of professional fulfillment among teachers and elevate the status of teaching as a respected profession. This study addresses a timely concern, given the increasing encouragement for teacher involvement in school decision-making in Chinese schools [8]. To date, empirical research on distributed leadership has primarily focused on its effects on teacher job satisfaction [9]. Moreover, most of this research has been conducted in Western contexts [10], while studies situated in the Chinese educational system remain limited. Findings across settings have also been inconsistent, partly due to contextual differences in how distributed leadership is enacted and perceived within varied institutional and cultural environments [11].

In parallel, growing scholarly attention has been directed toward teacher commitment and collaboration as critical factors influencing innovative practices. Research shows that teachers with strong professional commitment are more likely to engage in innovative pedagogical practices [12]. Regarding teacher collaboration, prior research has suggested that collaborative engagement among educators enhances their propensity to participate in innovative pedagogical practices. Teachers reported higher motivation, reduced workload, improved morale, efficiency, communication, and technological skills, and less personal isolation [13]. Meanwhile, distributed leadership supports teacher commitment by reducing stress, improving well-being, and promoting collaboration among teachers, ultimately creating a more supportive and engaged work environment [14]. When goal-oriented and supported by strong commitment, teacher collaboration enhances both innovative practices and professional development [15]. This suggests that distributed leadership may influence teachers’ innovative practices indirectly through teacher commitment and collaboration.

In China, centralized governance, collectivist values, and deeply embedded Confucian hierarchical norms have long constrained teacher participation in school-level decision-making. This cultural orientation not only shapes how leadership is enacted and perceived but may also be associated with variations in teachers’ autonomy and their engagement in pedagogical innovation. To address this gap, the present study examines the relationship between distributed leadership and teachers’ innovative practices in Chinese schools, with particular attention to the potential chain-mediating roles of teacher commitment and collaboration.

## 2. Literature review

### 2.1. Distributed leadership

While the school principal remains central to school development, teachers also play a pivotal role. This shift from individual to collective leadership is reflected in the concept of distributed leadership. Spillane [16] defines distributed leadership as a leadership practice emerging from the interactions among leaders, followers, and the situational context. Leadership is considered distributed when multiple individuals engage in collaboration and integrate their expertise to pursue common goals through coordinated actions [17,18]. A key aspect of distributed leadership is the active involvement of various stakeholders in decision-making processes. The TALIS 2018 data highlights the importance of schools offering opportunities for staff, students, and parents to participate in shared decision-making [19]. Research demonstrates that distributed leadership effectively fosters collaboration among teachers [20], promotes autonomy, and enhances instructional innovation [21]. However, other studies caution that excessive leadership intervention in daily instruction may undermine motivation and provoke resistance [22,23].

Despite its theoretical promise, the implementation of distributed leadership varies significantly across regions. According to Liu et al. [24], the distribution of leadership responsibilities among stakeholders is more common in Europe and South America, whereas schools in Asia and North America exhibit lower levels of leadership distribution. These regional differences suggest that the adoption of distributed leadership is not solely a matter of policy design, but is also influenced by broader contextual factors. As emphasized by Harris [25], the effectiveness of distributed leadership is deeply shaped by institutional structures and organizational culture.

In China, distributed leadership, as an imported concept, faces distinct contextual constraints. Two primary factors hinder its implementation. First, the country’s highly centralized educational governance tends to position teachers as passive recipients of top-down directives, limiting their involvement in school-level decision-making. This reflects a broader institutional tension between policy centralization and operational decentralization, which complicates the implementation of distributed leadership [26]. Second, deeply rooted cultural traditions shaped by Confucianism reinforce hierarchical norms and principal-centered authority, thereby constraining the development of shared and collaborative leadership practices [27].

Although research on distributed leadership in China has grown in recent years, empirical findings remain mixed. These inconsistencies may stem not only from structural and cultural barriers, but also from the absence or variability of key mediating mechanisms. For example, Liu et al. [9] found that distributed leadership was indirectly associated with teacher job satisfaction through autonomy and collaboration, but not through exchange and coordination. Similarly, Fan and Chu [26] identified teacher self-efficacy as a significant mediator, while teacher collaboration alone did not show a direct effect; however, both contributed to a sequential mediation model when combined. These differences suggest the need for greater theoretical and empirical attention to the distributed leadership effects in localized contexts.

### 2.2. Teacher commitment

Since the 1980s, Western researchers have shown increasing awareness of the complexity of teachers’ work and lives, prompting a growing focus on teacher commitment from the perspective of career development [28]. This perspective highlights the interaction among personal, institutional, and systemic contexts within a broader socio-historical landscape [29].

Researchers have proposed varying definitions of the dimensions of teacher commitment. In a comprehensive review of existing definitions, Firestone and Pennell [30] describe it as a psychological bond or sense of identification that an individual forms with an object that carries particular meaning and significance. More specifically, teacher commitment has been defined as teachers’ psychological attachment to the teaching profession, professional associations, schools, colleagues, parents, and students. As a key component of a school culture, it is manifested in teachers’ instructional practices, dedication to improving student outcomes, and loyalty to their school [31,32]. Although a unified definition of teacher commitment has yet to be established, it is typically characterized by personal, school-organizational, and educational system factors, as well as broader socio-historical contexts.

Teacher commitment, as a multidimensional construct, poses challenges for measuring its components. It is commonly categorized into three types: commitment to the teaching profession, to students, and to the school [33]. According to Abd Razak, Darmawan [34], teacher commitment can manifest in various forms, including commitment to the school, to student learning, to the work of teaching, and professionalism. Teacher commitment to the school reflects teachers’ engagement in behaviors that support the school’s goals, their willingness to exert effort beyond the basic requirements, and their intention to remain with the organization. In contrast, commitment to student learning denotes a teacher’s willingness to support the academic growth of low-achieving students. Commitment to the work of teaching refers to a teacher’s psychological identification with their role and their intention to actively invest in the teaching process. Lastly, commitment to professionalism involves an affective attachment to the teaching profession, associated with a social identification and satisfaction with the teaching role.

Moraal, Suhre [35] argued that teacher commitment comprises three components: affective commitment, normative commitment, and continuance commitment. They defined affective commitment as the desire to continue teaching and to find satisfaction in the work. Teachers with higher levels of affective commitment typically experience greater well-being, feel emotionally exhausted less frequently, exhibit reduced absenteeism, and demonstrate a lower intention to leave the profession. Normative commitment to the profession refers to the sense of moral obligation that employees feel to remain with an organization. This form of commitment arises when teachers perceive a moral responsibility toward their students or colleagues or when they recognize the substantial investment the organization has made in them, compelling them to reciprocate. Continuance commitment to the profession occurs when employees perceive a lack of alternative employment opportunities, leading them to continue working in their present employment due to the high perceived costs of leaving.

In this study, we adopted the definition of teacher commitment as outlined in the TALIS 2018. According to TALIS, teacher commitment is primarily reflected in dedication to both the profession and the school, encompassing attitudes toward the current job, professional development, and the teaching profession [19]. These findings notably align with the research conducted by Abd Razak, Darmawan [34], and by Moraal, Suhre [35] research.

### 2.3. Teacher collaboration

Teacher collaboration serves as an effective approach to building capacity, improving instructional practices, and enhancing student achievement [36]. Teacher collaboration includes cooperative professional development, peer support, co-teaching, sharing of instructional materials, and joint discussions on teaching and learning strategies [37,38]. Collaboration refers to the necessity for teachers to work collectively to accomplish specific instructional goals, develop competencies, and enhance student learning outcomes. The TALIS 2018 project explored the broad concept of teacher collaboration, including opportunities for interaction and involvement in instructional decision-making and practice [19]. In this study, teacher collaboration includes activities such as forming teaching teams within the same class, participating in joint activities across classes and grades, exchanging teaching materials, and collaborating with colleagues within the school [19].

### 2.4. Teachers’ innovative practices

The newly introduced *China’s Education Modernization 2035* outlines that “a highly qualified, professional, and innovative teaching workforce is key to accelerating educational modernization,” positioning “innovation” as a core competency for the teaching profession. A unified definition of teachers’ innovative practices has yet to be established. Gkorezis [39] defined teachers’ innovative practices as efforts to generate, promote, and implement ideas that aim to improve instructional quality. Teachers’ innovative practices are closely linked to their learning, social, educational, and technological competencies [40]. These practices are essential for enhancing educational effectiveness. Le Donné, Fraser [41] proposed three instructional approaches that significantly enhance student learning outcomes: active learning, cognitive activation, and teacher-directed instruction. Among these, the active learning method focuses on fostering student engagement in classroom activities through collaborative tasks, technology integration, and self-assessment. On the other hand, cognitive activation fosters learners’ ability to engage in critical thinking, problem-solving, and decision-making skills. These pedagogical methods are consistent with the innovative strategies outlined in the TALIS 2018 report [19]. This study focuses on teachers’ innovative instructional practices, defined as the intentional adoption of new instructional strategies, tools, or approaches to enhance student learning.

Among the factors influencing teachers’ innovative practices, distributed leadership emerges as a key catalyst. Some studies conducted in Chinese contexts using TALIS 2018 data offer converging evidence. One study [42], based on a sample of 2,451 senior high school teachers in Taiwan and using hierarchical linear modeling, examined the mediating roles of teacher autonomy and innovativeness in the relationship between distributed leadership and instructional quality. The results indicated that while distributed leadership negatively affected instructional quality directly, it enhanced teacher autonomy and innovativeness, both of which functioned as competitive partial mediators in this relationship. Another study [43] from Shanghai found that distributed leadership positively influenced teacher innovation. This effect was partially mediated by teacher collaboration and self-efficacy, with a chain mediation pathway observed from collaboration to self-efficacy. In addition, Fan and Chu [26] identified a similar sequential mechanism—distributed leadership enhancing teacher outcomes via collaboration and self-efficacy, albeit with job satisfaction as the outcome. While differing in focus, their findings support the plausibility of organizational and psychological mediators in linking leadership to teacher-level outcomes. Building on these findings, it is worthwhile to examine whether teacher commitment and collaboration function as sequential mediators in linking distributed leadership to teachers’ innovative practices within the Chinese context.

## 3. Chinese context

In February 2013, the Ministry of Education of mainland China published the first *Professional Standards for Compulsory Education School Principal* [44]. The standards emphasize that expanding decision-making participation shifts the role of teachers from “passive executors” to “active participants in governance.” Although these standards advocate for enhanced teacher participation, the hierarchical and centralized nature of China’s education governance not only continues to limit teachers’ roles in governance but also constrains the professional autonomy necessary for instructional innovation.

This governance structure not only suppresses innovation but also undermines motivational factors essential for sustained teacher engagement. China’s focus on teacher commitment is reflected in two primary concerns related to teaching and teacher policies: first, ensuring a high level of teacher effort to enhance school effectiveness; second, addressing teacher retention [45]. Within China’s high-stakes testing regime and rigid administrative framework, teachers are compelled to prioritize external accountability demands, which significantly undermines the development of commitment rooted in intrinsic motivation. Furthermore, in the underdeveloped regions of central and western China, teachers face challenges similar to those encountered in rural areas, particularly regarding retention and professional development [46].

In China, according to the directives of the Ministry of Education, Teaching Research Groups (TRGs), where educators who teach the same subject participate in daily activities of collective teaching and learning, serve as the primary platform for teacher collaboration. Under these cultural conditions, teachers are encouraged to engage in exchange pedagogical experiences related to lesson preparation, classroom management, and learning assessment, among other areas [47]. Teachers’ participation in TRGs significantly contributes to their professional identity development and facilitates their growth as educators [48]. Although TRGs facilitate professional development and foster collective practice, their heavily supervised and top-down structure [49] may also result in superficial collaboration, excessive formalization, and limited autonomy, especially in contexts characterized by intensive administrative inspections, excessive workloads, and limited institutional support [50,51].

## 4. Conceptual framework

**Fig 1:**
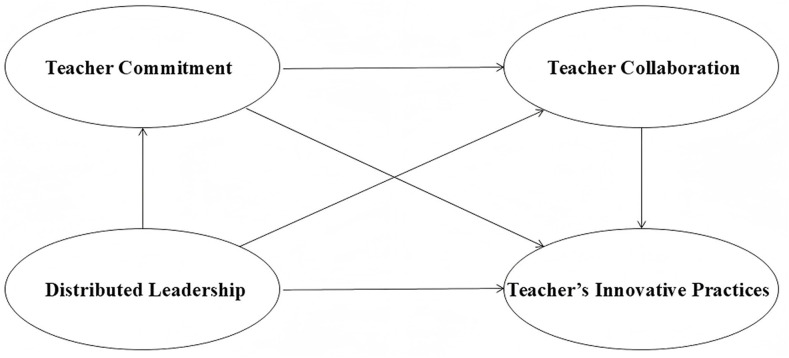
Conceptual framework.

The conceptual framework of this study is presented in Fig 1. Distributed leadership (DL) has attracted increasing attention in educational research, particularly for its potential to enhance teacher-level outcomes. A substantial body of empirical evidence has demonstrated that leadership enacted by school principals plays a pivotal role in promoting teacher commitment and collaboration [52,53]. These teacher-level processes, in turn, are closely associated with instructional innovation. For example, Firestone and Pennell [30] found that teacher commitment and collaboration are mutually reinforcing: collaborative interactions strengthen professional community and reduce isolation, thereby enhancing teachers’ sense of meaning in their work and motivating them to adopt innovative practices. In this study, innovative teaching practice is conceptualized as the intentional use of novel instructional methods, strategies, or materials aimed at enhancing student learning and engagement. It represents the outcome of sustained professional engagement, supported by teacher commitment and collaboration fostered through distributed leadership. Taken together, these observations underscore the importance of a robust theoretical framework to clarify the mechanisms through which distributed leadership fosters teacher commitment, collaboration, and ultimately instructional innovation.

To address this need, the study draws upon the Conservation of Resources (COR) theory [54], which posits that individuals are motivated to acquire, preserve, and invest resources that are personally or professionally valuable. Stress arises when these resources are threatened, lost, or insufficiently replenished. Within this framework, distributed leadership can be understood as a strategic resource orchestration mechanism. In the Chinese context, it operates through institutional roles such as teaching-research group leaders and subject coordinators, embedding collaboration into the organizational structure. These roles not only coordinate instructional tasks but also convert daily teacher interactions into resource pools for knowledge exchange and collective growth [55]. Such collaborative structures represent conditional resources that are shaped by organizational conditions and institutional arrangements. In contrast, teacher commitment reflects a trait-like resource, capturing the sustained professional identification and psychological investment that individuals bring to their work.

COR theory posits that conditional resources can interact with personal resources to form resource gain spirals [54]. Empirical evidence supports the plausibility of such resource-based mediation mechanisms. For instance, Fan and Chu [26] identified a chain mediation pathway involving self-efficacy and collaboration, through which distributed leadership influenced teacher job satisfaction, even within hierarchical systems. Drawing on COR theory, the present study proposes a chain mediation model in which distributed leadership is associated with teachers’ innovative practices through the sequential activation of teacher commitment and collaboration. In this framework, teacher commitment facilitates collaboration by fostering sustained professional engagement. When teachers feel highly committed, they are more likely to participate actively in collaborative activities, which in turn generate additional professional resources, including emotional energy, self-efficacy, and creative capacity [56]. Consistent with prior research [57–59], teacher commitment is expected to serve as a primary mediator, particularly under leadership characterized by accessibility and participatory empowerment [30], while teacher collaboration functions as a key mechanism for translating leadership into instructional improvement [60,61].

In summary, this study examined the associations between distributed leadership and teachers’ innovative practices, with particular attention to the potential mediating roles of teacher commitment and collaboration. Guided by this framework, the study addresses the following research questions:

(1) How is distributed leadership associated with teacher-related outcomes (e.g., teacher commitment, collaboration, and innovative practices) in China?
(2) Do teacher commitment and collaboration mediate the associations between distributed leadership and teachers’ innovative practices in China?

## 5. Methods

### 5.1. Data and samples

This study utilizes data from the 2018 cycle of the Teaching and Learning International Survey (TALIS) [19], a large-scale international assessment initiated by the OECD. The survey included 48 countries and regions, with nearly 260,000 participating teachers. In Shanghai, 3,976 teachers from 198 schools responded, yielding exceptionally high response rates—99.00% at the school level and 99.40% at the teacher level—well above the international benchmark of 75.00%. As a frontrunner in China’s educational reforms, Shanghai is widely considered to offer representative and high-quality teacher data. Given the study’s focus on distributed leadership, teacher commitment, collaboration, and instructional innovation, we screened the original dataset for completeness and relevance, resulting in 3,772 valid cases (valid response rate: 94.87%). Among the respondents, 74.30% were female and 25.70% male; 86.10% held a bachelor’s degree, 13.20% a master’s degree, and 0.70% a doctoral degree. This sample provides a robust foundation for subsequent analyses.

### 5.2. Variables

This section introduces the variables utilized in this study, which were derived from the TALIS 2018 teacher questionnaire. These variables are classified into three categories: the dependent variable, the independent variable, and the mediating variables (refer to S1 Table).

#### 5.2.1. Dependent variable.

The dependent variable in this study is teachers’ innovative instructional practices, which are narrowly defined as core teaching behaviors that occur in real time within the classroom, directly benefit students, and are subject to teachers’ personal control. This operationalization is grounded in two main considerations. First, the design of the TALIS 2018 questionnaire emphasizes observable, actionable, and cross-nationally comparable teaching behaviors, rather than broader curricular or systemic reforms. Second, within the Chinese context of basic education, the overall curriculum design or interdisciplinary program development is typically coordinated by school- or district-level teaching research teams, rather than by individual teachers. Therefore, focusing on classroom-level practices not only aligns with the original intent of TALIS but also better reflects the realities of teachers’ daily work in China. Based on this rationale, the study employs the following five items for measurement: (a) To what extent are you able to design high-quality questions for students in your teaching; (b) To what extent are you able to foster students’ critical thinking in your instruction; (c) To what extent are you able to implement diverse assessment strategies in your teaching; (d) To what extent are you able to flexibly adjust your instructional strategies during classroom practice; (e) To what extent are you able to utilize digital technologies (e.g., interactive whiteboards, computers, tablets) to support student learning. All items were rated on a 4-point Likert scale ranging from 1 (“Not at all”) to 4 (“To a great extent”). Higher scores indicate a higher level of teachers’ innovative instructional practices. The scale demonstrated good internal consistency, with a Cronbach’s alpha coefficient of 0.873.

#### 5.2.2. Independent variable.

The independent variable in this study is distributed leadership (DL), assessed using four items: (a) How strongly do you agree that this school offers staff opportunities to take part in school decisions actively; (b) How strongly do you agree that this school offers parents or guardians opportunities to take part in school decisions actively; (c) How strongly do you agree that this school offers students opportunities to take part in school decisions actively; (d) How strongly do you agree that this school has a shared-responsibility culture for school affairs. All items were assessed using a 4-point Likert scale ranging from 1 (“not at all”) to 4 (“a lot”). The reliability coefficient of DL was 0.933.

#### 5.2.3. Mediating variables.

The present study identified two mediating variables. The first, teacher commitment (CM), was measured using six items related to teachers’ feelings about their jobs: (a) I would transfer to another school if that were possible; (b) I would be satisfied with working at this school; (c) I would recommend this school as a desirable place to work; (d) Given the choice, I would still choose to become a teacher; (e) I regret of my decision to become a teacher; (f) I reflect on whether it would have been better to choose another profession. The items measuring teacher commitment were rated on a four-point Likert scale, where 1 indicates “strongly disagree,” 2 indicates “disagree,”3 indicates “agree,” and 4 indicates “strongly agree.” Items (a), (e), and (f) were negatively worded and thus reverse-coded prior to analysis. Higher scores indicate a stronger level of teacher commitment. The Cronbach’s alpha for this scale was 0.803, indicating good internal consistency.

The second mediating variable is teacher collaboration (CB), which consists of six items designed to assess collaborative practices among teachers. Respondents were asked, “On average, how often do you do the following in this school?” The six items included: (a) teaching jointly as a team in the same class; (b) observing other teachers’ classes and providing feedback; (c) engaging in joint activities across different classes and age groups; (d) exchanging teaching materials with colleagues; (e) engaging in discussions about the learning development of specific students; and (f) working with other teachers in this school to ensure common standards in evaluations for assessing student progress. The items were set with six options: “never,” “once a year or less,” “two to four times a year,” “five to ten times a year,” “one to three times a month,” and “once a week or more.” These options were rated using a six-point Likert scale, scored from 1 to 6. The alpha coefficient for this scale was 0.815, indicating good internal consistency.

### 5.3. Analysis procedures

Data analysis for this study was conducted using SPSS 24.0 and AMOS 26.0 software. First, the data were assessed for Common Method Bias (CMB), and the measurement scale’s validity was evaluated through three dimensions: structural validity, convergent validity, and discriminant validity., followed by descriptive statistics and correlation analyses Second, a structural equation model (SEM) was constructed using AMOS 26.0 to examine the direct relationships among distributed leadership, teacher commitment, teacher collaboration, and teachers’ innovative practices, based on the predefined hypotheses. Model fit was assessed using the following indices: Comparative Fit Index (CFI), Goodness-of-Fit Index (GFI), Adjusted Goodness-of-Fit Index (AGFI), Tucker-Lewis Index (TLI), and Normed Fit Index (NFI), all of which exceeded 0.90. Additionally, the Root Mean Square Error of Approximation (RMSEA) was below 0.08, and the Standardized Root Mean Square Residual (SRMR) was below 0.05 [62]. Third, this study employed the Bootstrap method to assess the mediating effect, utilizing 5,000 randomly selected bootstrap samples with a 95% confidence interval [63]. Furthermore, the study compared the fit indices of various structural equation models—including the full mediation model and the partial mediation model—to assess the robustness of the chained mediation model.

## 6. Results

### 6.1. Testing and control of common method bias

Given that the TALIS 2018 survey involved multiple thematic questionnaires administered to the same respondents, there is a potential risk of common method bias that could compromise the validity of the measures. Therefore, it is essential to assess common method bias to ensure the reliability of the research findings. To this end, this study used Harman’s single-factor test statistical control. Harman’s single-factor test revealed four factors with eigenvalues greater than 1. The first factor explained 29.816% of the total variance, which is below the commonly accepted threshold of 40%, suggesting minimal common method bias. In summary, this study does not exhibit significant common method bias, and the subsequent descriptive analysis, correlation analysis, and effect testing yielded statistically significant results.

### 6.2. Validity testing and interpretation of results

**Fig 2:**
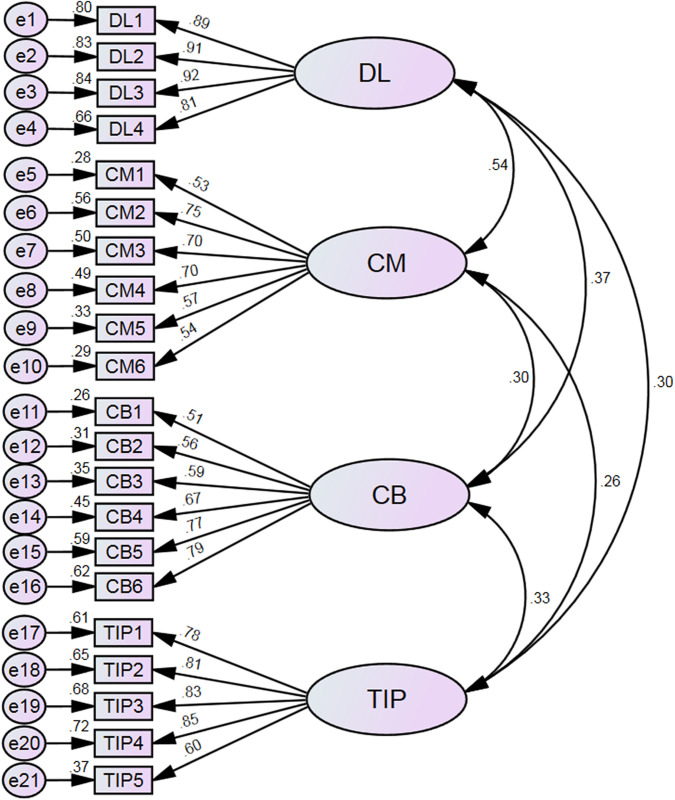
Structural validity assessment of the model.

First, confirmatory factor analysis (CFA) was conducted to examine the correspondence between observed variables and latent constructs, resulting in the model depicted in Fig 2. The model yielded satisfactory fit indices: CFI = 0.938, GFI = 0.932, AGFI = 0.914, TLI = 0.929, NFI = 0.934, RMSEA = 0.059, and SRMR = 0.042. These values exceed commonly accepted thresholds (i.e., CFI, GFI, AGFI, TLI, and NFI ≥ 0.90; RMSEA and SRMR ≤ 0.08), indicating that the model is adequately specified and demonstrates acceptable overall fit [64,65].

Second, convergent validity was assessed using factor loadings, average variance extracted (AVE), and composite reliability (CR). The factor loadings for distributed leadership ranged from 0.813 to 0.916, with an AVE of 0.782 and a CR of 0.935. For teacher commitment, factor loadings ranged from 0.525 to 0.750, the AVE was 0.408, and the CR was 0.802. For teacher collaboration, the factor loadings ranged from 0.507 to 0.789, with an AVE of 0.430 and a CR of 0.815. For teachers’ innovative practices, factor loadings ranged from 0.604 to 0.846, the AVE was 0.604, and the CR was 0.883. These results indicate that the observed items are highly representative of their respective latent constructs, demonstrating acceptable levels of convergent validity overall.

Finally, the heterotrait–monotrait (HTMT) ratio was employed to assess discriminant validity. As shown in Table 1, the HTMT value between distributed leadership and teacher commitment was 0.546; between distributed leadership and teacher collaboration, 0.370; and between distributed leadership and innovative instructional practices, 0.297. The HTMT values between teacher commitment and teacher collaboration, teacher commitment and innovative instructional practices, and teacher collaboration and innovative instructional practices were 0.300, 0.259, and 0.336, respectively. All HTMT values were below the recommended threshold of 0.850, as proposed by Hair et al. [66], indicating satisfactory discriminant validity among the latent constructs.

**Table 1:** Results of the Heterotrait–Monotrait Ratio (HTMT) test.

Latent Variable	DL	CM	CB	TIP
DL	——			
CM	0.546	——		
CB	0.370	0.300	——	
TIP	0.297	0.259	0.336	——

Taken together, the results indicate that the measurement scales demonstrate satisfactory validity and can effectively assess the status of distributed leadership in schools, the level of teacher commitment, the extent of teacher collaboration, and the quality of innovative instructional practices.

### 6.3. Descriptive statistics and correlations

Table 2 demonstrates descriptive statistics for each latent variable. The mean scores for distributed leadership (3.003), teacher commitment (2.870), teacher collaboration (3.569), and teachers’ innovative practices (3.205) were above the median, suggesting moderately high levels of these constructs. The median values for distributed leadership, teacher commitment, and teachers’ innovative practices are all 2.5, while the median for teacher collaboration is 3.5. These figures provide an important basis for subsequent comparative analyses. Pearson product-moment correlation analyses were conducted among the variables in this study. The results indicated that the correlation coefficients between distributed leadership and teacher commitment, teacher collaboration, and teachers’ innovative practices were r = 0.545 (p < .001), r = 0.369 (p < .001), and r = 0.297 (p < .001), respectively. The correlation coefficients between teacher commitment and teacher collaboration, and teacher commitment and teachers’ 2.5innovative practices were r = 0.298 (p < .001) and r = 0.258 (p < .001), respectively. The correlation coefficient between teacher collaboration and teachers’ innovative practices was r = 0.335 (p < .001).

**Table 2:** Descriptive statistics and correlation analysis of variables.

Variables	Med	M	SD	1	2	3	4
1.DL	2.500	3.003	0.623	——			
2.CM	2.500	2.870	0.495	0.545***	——		
3.CB	3.500	3.569	1.022	0.369***	0.298***	——	
4.TIP	2.500	3.205	0.571	0.297***	0.258***	0.335***	——

Note: DL = Distributed Leadership; CM = Teacher Commitment; CB = Teacher Collaboration; TIP = Teachers’ Innovative Practices; ***p < .001.

### 6.4. Mediation effect testing of distributed leadership on teachers

**Fig 3:**
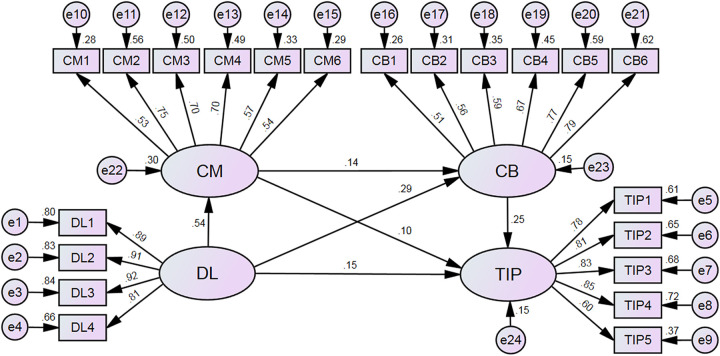
Chained mediation model of teacher commitment and teacher collaboration (Standardized).

To further elucidate the relationships among distributed leadership, teacher commitment, teacher collaboration, and teachers’ innovative practices, this study utilized AMOS 26.0 to construct a chain mediation model, which is shown in Fig 3, based on the predefined hypotheses. The model was subsequently tested, with a detailed comparison of the model fit indices and corresponding parameter estimates. The results demonstrate that all model fit indices—CFI (0.938), GFI (0.932), AGFI (0.914), TLI (0.929), NFI (0.934), RMSEA (0.059), and SRMR (0.0418)—indicate a good fit. These values suggest that the fit indices meet acceptable standards. Therefore, it can be concluded that the study data align well with the hypothesized model, lending credibility to the model’s results.

Table 3 presents the results of the validity assessment. In the chained mediation model involving distributed leadership, teacher commitment, teacher collaboration, and teacher innovative practices, all path coefficients reached a high level of significance (p < 0.001). Specifically, the direct effect of distributed leadership on teacher innovative practices was significant, with a path coefficient of 0.149 (CR = 6.840); the path coefficient from distributed leadership to teacher commitment was 0.545 (CR = 23.447); from teacher commitment to teacher innovative practices was 0.103 (CR = 4.527); from distributed leadership to teacher collaboration was 0.294 (CR = 12.464); from teacher collaboration to teacher innovative practices was 0.249 (CR = 11.892); and from teacher commitment to teacher collaboration was 0.138 (CR = 5.828). These findings indicate that distributed leadership exerts significant positive effects not only on teacher commitment and teacher collaboration, but also directly on teacher innovative practices. Furthermore, teacher commitment positively influences both teacher collaboration and innovative practices, while teacher collaboration also significantly predicts teacher innovative practices. Overall, the empirical results provide strong support for all six hypotheses proposed in Table 3, confirming the theoretical soundness and empirical fit of the proposed model.

**Table 3:** Results of main effect testing.

Research Hypotheses	Path Relationships	Standardized Path Coefficients	C.R.	P	Validation of Hypotheses
H1	DL → TIP	0.149	6.840	***	Validation
H2	DL → CM	0.545	23.447	***	Validation
H3	CM → TIP	0.103	4.527	***	Validation
H5	DL → CB	0.294	12.464	***	Validation
H6	CB → TIP	0.249	11.892	***	Validation
H8	CM → CB	0.138	5.828	***	Validation

To further investigate the specific mechanisms by which distributed leadership is associated with teachers’ instructional innovation capabilities, this study employed the Bootstrap method, utilizing 5,000 Bootstrap samples drawn repeatedly at random, with the confidence interval set at 95%. A significant mediation effect is indicated if the confidence intervals for the path coefficients do not contain zero [67]. The indirect effect for Path 1 is 0.061, with an absolute Z value of 4.357, which exceeds the 1.96 threshold. Furthermore, both the Bias-Corrected and Percentile 95% Confidence Intervals (CIs) for Path 1 do not include zero, confirming the presence of a statistically significant mediation effect for this path. Similarly, the mediation effects for Paths 2 and 3 are significant as well. Thus, teacher commitment and teacher collaboration serve as independent mediators between distributed leadership and teachers’ innovative practices. Additionally, the pathway “teacher commitment → teacher collaboration” acts as a chained mediator in this relationship.

The direct effect of distributed leadership on teachers’ innovative practices accounts for 50.15% of the total effect, whereas the mediating effect contributes 49.85%. Additionally, this study further explores the impact of different mediation chains by comparing the coefficients of the indirect mediation effects. As shown in Table 4, the mediation effect of Path 1 accounts for 37.89%, Path 2 for 49.07%, and the chained mediation effect of Path 3 for 13.04% of the total indirect effect. These findings indicate that teacher collaboration demonstrates the strongest mediating effect, followed by teacher commitment, while the chained mediation effect of “teacher commitment → teacher collaboration” is comparatively weaker.

**Table 4:** Results of mediation effect testing.

Path	Point Estimate	Product of Coefficients		Bootstrapping			
				Bias-Corrected95% CI		Percentile 95% CI	
		SE	Z	Lower	Upper	Lower	Upper
Path 1. DL → CM → TIP	0.061	0.014	4.357	0.035	0.090	0.034	0.088
Path2.DL → CB → TIP	0.079	0.009	8.778	0.063	0.099	0.062	0.098
Path3.DL → CM → CB → TIP	0.021	0.004	5.250	0.014	0.028	0.013	0.028
Total Indirect Effect	0.161	0.017	9.471	0.132	0.196	0.127	0.193
Direct Effect	0.162	0.026	6.231	0.110	0.213	0.112	0.215
Total Effect	0.322	0.022	14.636	0.280	0.365	0.281	0.365

### 6.5. Robustness check

This study constructed four structural equation models: a full mediation model, partial mediation model 1, partial mediation model 2, and partial mediation model 3 (refer to S2 Fig). The fit indices were compared to assess the robustness of the chained mediation model, with the results detailed in Table 5. The comparison revealed that, except for the SRMR value, all fit indices met ideal standards, with the full mediation model showing a relatively poorer fit (0.930, 0.925, 0.907, and 0.926) and the chained mediation model showing the best fit (0.938, 0.932, 0.914, and 0.934). These findings suggest that the models developed in this study are robust and offer a plausible explanation of how distributed leadership may be linked to teachers’ instructional innovation capabilities.

**Table 5:** Comparison of model fit indices.

Model	CFI	GFI	AGFI	TLI	NFI	RMSEA	SRMR
Full Mediation Model	0.930	0.925	0.907	0.921	0.926	0.063	0.0776
Partial Mediation Model 1	0.933	0.927	0.909	0.924	0.929	0.061	0.0573
Partial Mediation Model 2	0.934	0.928	0.909	0.924	0.929	0.061	0.0567
Partial Mediation Model 3	0.938	0.931	0.914	0.929	0.933	0.060	0.0435
Chained Mediation Model	0.938	0.932	0.914	0.929	0.934	0.059	0.0418

## 7. Discussion

This study demonstrates that distributed leadership is meaningfully associated with teacher commitment, collaboration, and innovative practices. This research focused on how distributed leadership may contribute to teachers’ innovative practices, with particular attention to the potential mediating roles of teacher commitment and collaboration. The principal findings are presented below.

First, the study reveals that distributed leadership is positively associated with teachers’ innovative practices, aligning with prior research of O’Shea [68], which views it as an educational philosophy conducive to fostering innovation. Distributed leadership provides teachers with decision-making autonomy regardless of their formal status, enabling them to implement instructional strategies that effectively prepare students for the future. As noted by Spillane, Halverson [69], educational innovation often relies on creative organizational structures and decentralized leadership. Expanding school autonomy may further facilitate innovation [70]. These findings are also consistent with [63] Amels, Krüger [71], who reported that teachers’ perceptions of distributed leadership were linked to greater willingness to contribute to school development and a stronger perceived capacity to engage in educational reform. While DL may be limited in hierarchical systems [18], our findings show it can still be effective in China. Despite centralized control, schools often allow informal collaboration through teaching-research groups [55]. These structures support trust and commitment, which may, according to COR theory [54], correlate with innovation by reinforcing professional engagement. This suggests DL’s impact depends less on system type and more on how internal resources are mobilized.

Second, this study revealed that teacher commitment mediates the relationship between distributed leadership and innovative practices. Supportive principals positively influence teachers’ commitment by providing feedback, encouragement, recognition, and setting clear objectives [72,73]. When collective efficacy is embedded in school culture, distributed leadership helps shape teachers’ shared beliefs and positive attitudes. Joo [74] demonstrated that teachers’ collective beliefs influence professionalism by fostering collaborative school conditions and enhancing positive emotions. Hulpia and Devos [75] argue that the leadership team’s collaboration significantly influences organizational commitment. Teachers are more likely to feel committed when leaders are accessible, empowering, and responsive.

Furthermore, teacher collaboration also mediates the relationship between distributed leadership and innovation. Prior research has shown that distributed leadership fosters teacher connectivity and collegial interaction [76]. This empowerment, brought about by distributed leadership, may support increased collaboration among educators. When autonomy is supported, teachers participate more actively in shared decision-making, which in turn enhances innovative teaching. Lin [77] noted that collectivism in teaching fosters professional networks and shared innovation. Teachers who feel a sense of belonging to their professional community are more willing to exchange ideas and practices. While TRGs have been critiqued for superficial collaboration [78], this paradox may reflect contextual variation. In schools where distributed leadership is meaningfully practiced, TRGs can become platforms for genuine professional learning [55]. From a COR perspective [56], such environments -when fostering trust and shared resources – can promote innovation by enhancing individuals’ resource security and professional engagement. Our findings suggest that the value of TRGs depends not on structure alone, but on how school leaders activate internal capacities for authentic collaboration.

Finally, this study found that the chained mediation of “teacher commitment → teacher collaboration” serves as a crucial mechanism through which distributed leadership enhances innovative practices. de Jong, de Kleijn [79] found that distributed leadership fosters a stronger collaborative orientation focused on collective educational improvement. Through collaboration, self-evaluation, and feedback, teachers refine their practices and support ongoing professional growth. Distributed leadership fosters a sense of ownership and responsibility, which in turn boosts commitment and deepens collaboration—critical ingredients for innovation.

Based on the findings, we propose the following recommendations. First, school principals should empower middle-level administrators—such as department heads or teaching-research group leaders—by delegating instructional leadership responsibilities, involving them in school-level planning, and supporting their professional development [80]. Second, school leaders should enhance teacher commitment by offering both material and emotional support, reducing work-related stress, and fostering a harmonious and participatory school climate. Prioritizing teachers’ overall well-being can strengthen their sense of belonging and intrinsic motivation, thereby encouraging greater engagement in innovative teaching practices. Third, to strengthen teacher collaboration, school leaders should move beyond informal interactions and design purposeful, structured opportunities [81]. To reduce the superficiality of TRGs, principals can participate in TRG sessions and provide feedback to improve focus and accountability. Adopting a thematic discussion model and encouraging cross-grade or interdisciplinary exchanges can promote coherence and innovation. Moreover, building mechanisms to implement and reflect on TRG outcomes can help translate discussion into tangible teaching improvements.

## 8. Limitations and prospects

This study explored the mechanisms through which distributed leadership relates to teachers’ instructional innovation by integrating theoretical reasoning, research design, and empirical testing. However, several limitations warrant attention and offer directions for future research. First, future studies may incorporate classroom observations, student evaluations, or administrative records to enable data triangulation and mitigate the limitations associated with cross-sectional and self-reported data. Second, the current dataset captures only the frequency of teacher collaboration, without addressing its qualitative dimensions—such as trust, depth, or shared goals. To gain a deeper understanding of the nature and quality of collaboration, future research could adopt qualitative approaches, including interviews, focus groups, or classroom ethnography. Third, to maintain model parsimony, this study did not control for key covariates such as teaching experience, school type, or urban–rural location. Future studies could employ multi-group structural equation modeling or multilevel modeling to examine potential heterogeneity across teacher and school characteristics. Finally, although this study highlights teacher commitment and collaboration as key mediators based on COR theory, we acknowledge that teacher autonomy may also play an important role. Distributed leadership can enhance autonomy, which in turn supports instructional innovation [82]. Future research could examine autonomy as an alternative or complementary mediator to better capture the full range of internal resources mobilized under distributed leadership.

## Supporting information

S1 Table Items measuring distributed leadership, teacher commitment, teacher collaboration, and teachers’ innovative practices. (PDF)

S2 Fig Robustness check model. (TIF)
