# Supplementary material for: Linking distributed leadership to teachers’ innovation: Chain mediating roles of commitment and collaboration in Chinese schools
Source: PLoS One. 2025 Sep 24;20(9):e0333118. doi: 10.1371/journal.pone.0333118 (PMC12459820; doi:10.1371/journal.pone.0333118)
Supplement: S1 Table — (PDF) [file pone.0333118.s001.pdf]

Supporting information

S1 Table. Items Measuring Distributed Leadership, Teacher Commitment, Teacher Collaboration, and Teachers' Innovative Practices

Construct	Item wording	Item coding
Distributed Leadership	How strongly do you agree that this school offers staff opportunities to take part in school decisions actively?	DL1
	How strongly do you agree that this school offers parents or guardians opportunities to take part in school decisions actively?	DL2
	How strongly do you agree that this school offers students opportunities to take part in school decisions actively?	DL3
	How strongly do you agree that this school has a shared-responsibility culture for school affairs?	DL4
Teacher Commitment	I would transfer to another school if that were possible?	CM1
	I would be satisfied with working at this school?	CM2
	I would recommend this school as a desirable place to work?	CM3
	Given the choice, I would still choose to become a teacher?	CM4
	I regret of my decision to become a teacher?	CM5
	I reflect on whether it would have been better to choose another profession?	CM6
Teacher Collaboration	How often do you teach jointly as a team in the same class?	CB1
	How often do you observe other teachers` classes and provide feedback?	CB2
	How often do you engage in joint activities across different classes and age groups?	CB3
	How often do you exchange teaching materials with colleagues?	CB4
	How often do you engage in discussions about the learning development of specific students?	CB5
	How often do you work with other teachers in this school to ensure common standards in evaluations for assessing student progress?	CB6
Teachers' Innovative Practices	To what extent are you able to design high-quality questions for students in your teaching?	TI1
	To what extent are you able to foster students' critical thinking in your instruction?	TI2
	To what extent are you able to implement diverse assessment strategies in your teaching?	TI3
	To what extent are you able to flexibly adjust your instructional strategies during classroom practice?	TI4
	To what extent are you able to utilize digital technologies (e.g., interactive whiteboards, computers, tablets) to support student learning?	TI5
