# Supplementary figures and images for: Linking distributed leadership to teachers’ innovation: Chain mediating roles of commitment and collaboration in Chinese schools
Source: PLoS One. 2025 Sep 24;20(9):e0333118. doi: 10.1371/journal.pone.0333118 (PMC12459820; doi:10.1371/journal.pone.0333118)

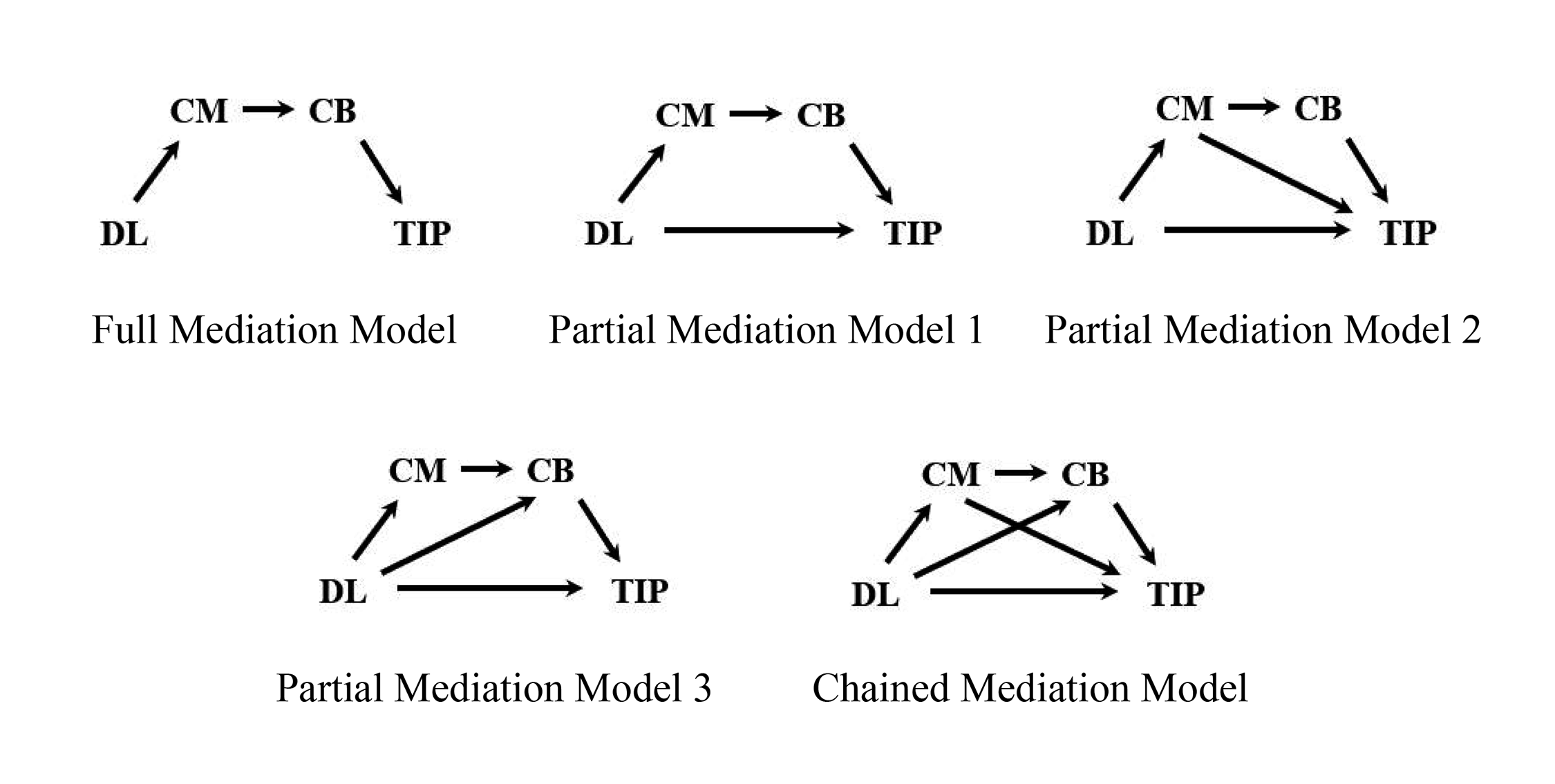

Supplement: S2 Fig — (TIF) [file pone.0333118.s002.tif]
